# Carpal tunnel syndrome and exposure to work-related biomechanical stressors and chemicals: Findings from the Constances cohort

**DOI:** 10.1371/journal.pone.0235051

**Published:** 2020-06-25

**Authors:** Yves Roquelaure, Ronan Garlantézec, Vincent Rousseau, Alexis Descatha, Bradley Evanoff, Stefano Mattioli, Marcel Goldberg, Marie Zins, Julie Bodin

**Affiliations:** 1 Univ Angers, CHU Angers, Univ Rennes, Inserm, EHESP, Irset (Institut de recherche en santé, environnement et travail) - UMR_S 1085, Angers, France; 2 Univ Rennes, CHU Rennes, Inserm, EHESP, Irset (Institut de recherche en santé, environnement et travail) - UMR_S 1085, Rennes, France; 3 INSERM UMS 011, Population Based Epidemiological cohorts Unit and Paris Descartes University, Paris, France; 4 Division of General Medical Sciences, Washington University School of Medicine, St. Louis, Missouri, United States of America; 5 Department of Medical and Surgical Sciences, Occupational Medicine, University of Bologna, Bologna, Italy; Ohio State University, UNITED STATES

## Abstract

**Objective:**

To investigate the effects of co-exposure to biomechanical wrist stressors and chemicals on the risk of CTS in a large cohort of French workers.

**Methods:**

Prospective study using the data collected at baseline and at the first 12 month-follow-up for the 18,018 participants included in the population-based Constances cohort between 2012 and 2015. CTS at follow-up and exposure to biomechanical wrist stressors and chemicals at baseline were assessed using a self-administered questionnaire. Associations between CTS and co-exposure to biomechanical wrist stressors and chemicals were studied using multivariate logistic regression models, adjusted for personal/medical factors.

**Results:**

184 men (2.1%, 95%CI 1.8–2.4) and 331 women (3.6%, 3.2–3.9) free from chronic hand symptoms at baseline declared suffering from unilateral/bilateral CTS at follow-up. A potentiating effect of co-exposure to biomechanical wrist stressors and chemicals on the risk of CTS was found for both genders, with higher OR in the co-exposure group (OR = 3.38 [2.29–5.01] in men and OR = 4.12 [2.73–6.21] in women) than in the biomechanical exposure group (OR = 2.14 [1.51–3.03] in men and OR = 2.19 [1.72–2.78] in women) compared to no exposure group.

**Conclusions:**

The study showed an association between CTS and co-exposure to biomechanical wrist stressors and chemicals, after adjustment for the main personal and medical factors. This finding should be confirmed using more objective case definition of CTS and assessment of the chemical exposure before drawing conclusions on the possible synergistic effects of mechanical stressors and chemical on the median nerve.

## Introduction

Carpal tunnel syndrome (CTS) is the most common entrapment neuropathy in the general population as well as the working population [1]. CTS is a leading cause of surgery and occupational diseases (OD) in many industrialized countries [1,2].

CTS is responsible for numbness, tingling, or burning sensation, and/or pain in the median nerve innervated areas of the hand. CTS could lead to decreased digit force production and dexterity impairing precision pinch movements and fine manual tasks during working and daily activities [3].

Certain personal characteristics (e.g., age, gender) [4,5] and medical conditions (e.g., obesity, diabetes mellitus, arthritis) [6–8] increase the risk of CTS. Working conditions exposing workers to biomechanical stressors are known to increase the incidence of CTS, namely repetitive movements, hand-arm transmitted vibration, and forceful manual exertion [1,9–14]. The relationships between CTS and work-related psychological stressors, such as job stain, are less well established [15,16]. In contrast to personal and medical risk factors for CTS, occupational risk factors can be modified by preventive interventions in the workplace.

Most studies on CTS focused on the personal factors and work-related exposure to biomechanical and psychosocial stressors, while workers are often co-exposed to biomechanical stressors, psychosocial factors, and chemical agents in the real working conditions. This is particularly true in some occupations and industry sectors at high risk for CTS (e.g., construction and cleaning sectors), and justifies an integrative approach of the work exposure [17,18]. We still lack epidemiological data on the possible impact of chemical exposure on the risk of CTS, despite the potential neurotoxic effects of some chemicals (e.g., n-hexane) [19–22]. The frequent biomechanical and chemical co-exposure [23] raises the question of potential synergistic effects of mechanical stressors and neurotoxic chemicals on the risk of CTS according to several putative mechanisms. Exposure to chemicals may generate diffuse subtle nerve damage rendering the median nerve more prone to entrapment at the carpal tunnel. This may potentiate, as is suggested for diabetic polyneuropathy, the effects of mechanical stress during tasks exposing to both biomechanical wrist stressors and neurotoxic chemicals [24,25].

A scoping review executed through PubMed on November the 9th 2019, using the MeSH Term “carpal tunnel syndrome” and some terms referring to neurotoxic agents (chemical substance* OR insecticide* OR lead poisoning [MH] OR neurotoxi* OR particulate OR pesticide* OR pollution OR solvent* OR toxic), permitted us to retrieve 68 citations. Out of these citations, 6 articles seemed to be pertinent in some way. The oldest one was by Bleecker in 1986, the author proposed to examine the vibration perception of workers co-exposed to neurotoxic agents and to biomechanical overload of hand and forearm, with the aim to consider the possible co-presence of damages due to chemical substances and to nerve compression [26]. Among the others papers, two were regarding co-exposure to pesticides [27,28], one was concerning the low-level chronic exposure to lead [29], one was on Toxic Oil Syndrome [30], and the last-one regarded particulate matter [31].

The present study aimed to investigate prospectively the effects of co-exposure to biomechanical wrist stressors and chemicals on the risk of CTS in a large cohort of French workers. The main hypothesis of the study was a potentiating effect of co-exposure to biomechanical wrist stressors and potentially neurotoxic chemicals on the risk of CTS.

## Methods

### Study population

The Constances study was approved by authorities regulating ethical data collection in France (CCTIRS: Comité Consultatif pour le Traitement des Informations Relatives à la Santé; CNIL-Commission Nationale Informatique et Liberté) and all participants signed an informed consent.

The Constances cohort is a national population-based cohort of randomly recruited participants, including volunteers aged 18–69 years at baseline recruited in 22 selected Health Screening Centers (HSC) from the principal regions of France [32]. Using a random sampling strategy stratified according to unequal inclusion probabilities, participants were invited to participate, with a response rate of 7.3% [32]. At inclusion, the selected subjects are invited to complete questionnaires and to attend a HSC for a comprehensive health examination. The follow-up includes a yearly self-administered questionnaire [32].

### Data

The present study used the data collected by questionnaire at baseline and at the first 12 month-follow-up for the 39,487 participants included between 2012 and 2015 (S1 Fig and S1 File). We excluded subjects with the following characteristics at baseline: 1) participants professionally non active at baseline (n = 14,240), 2) self-declared CTS or chronic hand symptoms at baseline (n = 2,295), 3) pregnant women at follow up (n = 333) and 4) workers with missing data for at least one of the variables studied (n = 4,601). Finally, 18,018 persons (8,733 men and 9,285 women) with complete data have been included in the study.

### Risk factors at baseline

#### Personal and medical risk factors

Age was dichotomized in two classes (< 45 yrs, ≥ 45 yrs). Body mass index (BMI) was categorized in three classes (< 25, ≥ 25–30, ≥ 30 Kg/m^2^). The information on diabetes mellitus and rheumatoid arthritis (whether requiring prescription drugs or not) was grouped in a new variable (medical problems) due to the small number of cases. Alcohol use disorders (Alcohol Use Disorders Identification Test (AUDIT-C)) [33] were self-assessed.

#### Work-related psychosocial factor

Effort–reward imbalance (ERI) was assessed at baseline using the French short version of the Siegrist’s questionnaire [34].

#### Work-related biomechanical stressors

Biomechanical exposure in the 12 month period preceding baseline was assessed by questionnaire using definitions of the European criteria document for the relatedness of MSDs [35].

“*Biomechanical wrist exposure”* was defined as exposure to at least one of the following factors:

1. *High biomechanical perceived exertion* (score ≥12 on the Borg rating of Perceived Exertion scale (20-RPE), graduated from 6 (‘very, very light’) to 20 (‘maximum exertion’)).
2. *Repetitive hand movements* (performing more than two actions per minute for more than 4 hours / day),
3. *Hand-transmitted vibrations* (use of a vibrating hand-tool for more than 2 hours / day),
4. *Awkward wrist postures* (repetitive or sustained wrist bending for more than 2 hours / day),
5. *Repetitive pinching* (holding tools/objects in a pinch grip for more than 4 hours / day),

#### Work-related exposure to chemicals

Six potentially neurotoxic chemical product groups were identified among the 36 chemicals (generic names) and mixtures of chemicals under study according to several classification methods of neurotoxicity [18–22,36,37]: trichloroethylene, white spirit (mineral spirits), cellulosic diluent, paints and varnishes, inks and dyes, and pesticides (weed killers, insecticides, fungicides). Chemical exposure to at least one of the six potentially neurotoxic chemicals was assessed for each respondent’s entire occupational life.

An *exposure* variable to biomechanical wrist stressors and chemicals was created according to the four following categories:

1. *No exposure group*: no exposure to any of the five biomechanical wrist stressors and no exposure to any of the six chemicals,
2. *Chemical exposure group*: only exposure to at least one of the six chemicals,
3. *Biomechanical exposure group*: only exposure to at least one of the five biomechanical wrist constraints,
4. *Co-exposure group*: exposure to both biomechanical wrist stressors (at least one of the five) and chemicals (at least one of the six).

### Outcome at follow-up

At the 12-month follow-up, 49 health problems were self-assessed via a self-administered questionnaire. CTS was assessed by answering the question: “*Do you suffer or have you suffered from CTS in the last 12 months (whether CTS required sick leave or not and/or treatment or no*t)?”.

### Statistical analyses

Comparison between men and women were studied by Chi-squared or Fisher’s exact tests.

Univariate logistic regression analyses were run to test the associations between the occurrence of CTS and potential risk factors at baseline in men and women separately [38]. Then, five multivariate logistic regressions were performed:

- *Model 1*: Including personal and medical risk factors; all personal and medical factors were forced into the models because of their well-known association with CTS in the literature (e.g. age, BMI, medical problems, current alcohol consumption). High effort-reward imbalance (ERI ratio > 1) was retained in the models because of association with CTS (p<0.20) in the univariate analysis.
- *Model 2*: Biomechanical wrist exposure added to model 1;
- *Model 3*: Chemical exposure added to model 1;
- *Model 4*: Exposure to biomechanical wrist stressors and chemical exposure including a co-exposure group added to model 1 to assess the effect of co-exposure.

Interaction between biomechanical wrist exposure and chemical exposure was tested and was not significant (p = 0.300 in men and p = 0.205 in women). A sensitivity analysis was performed for a subpopulation restricted to low-grade white-collar workers and blue-collar workers. All analyses were performed using SAS 9.4 using the logistic procedure. Results are presented as Odds ratio (OR) with their 95% confidence intervals (95% CI). A p value <0.05 was considered as statistically significant.

## Results

### Participant characteristics

A description of the 8,733 men and 9,285 women is given in Table 1. The majority of men were managers, professionals (48.9%), and professional or technical workers (25.6%) and, to a lesser extent, blue collar workers (15.0%) and lower-level white-collar workers (8.9%). The majority of women were professional or technical workers (38.4%), managers, teachers (30.9%), and lower-level white-collar workers (27.7%); few women were blue-collar workers (2.4%) (Table 1).

**Table 1:** Personal/medical factors and exposure to work-related factors in men and women.

	Men (N = 8,733)		Women (N = 9,285)		p^a^
	n	%	n	%	
**Personal/medical factors**					
Self-declared CTS	184	2.1	331	3.6	**<0.001**
Age 45 or over (yrs)	4,598	52.7	4,571	49.2	**<0.001**
Diabetes and/or rheumatoid arthritis	222	2.5	189	2.0	**0.023**
Body mass index (BMI, kg/m^2^)					**<0.001**
Lean/normal < 25	4,614	52.8	6,529	70.3	
Overweight [25–30]	3,316	38.0	1,901	20.5	
Obesity ≥ 30	803	9.2	855	9.2	
Alcohol consumption					**<0.001**
Abstinence	403	4.6	743	8.0	
Consumption without risk	2,954	33.8	5,655	60.9	
Consumption with low risk	4,505	51.6	2,519	27.1	
Alcohol use disorders	871	10.0	368	4.0	
Occupational category (Nmiss: 2459)					**<0.001**^b^
1—Farmers	2	0.0	2	0.0	
2 –Craftsmen, salesmen and managers	109	1.7	49	0.6	
3 –Professionals	3,224	48.8	2,340	30.9	
4—Technicians and associate professionals^c^	1,691	25.6	2,914	38.4	
5 –lower-level white-collar workers	585	8.9	2,097	27.7	
6 –Blue collar workers^d^	989	15.0	183	2.4	
**Work-related psychosocial factor**					
Effort-reward imbalance ratio >1	4,051	46.4	4,762	51.3	**<0.001**
**Work-related biomechanical stressors**					
Biomechanical exposure (at least one of the following factor)	3,309	37.9	3,911	42.1	**<0.001**
High physical perceived exertion (RPE ≥12) (Nmiss: 65)	2,450	28.2	2,665	28.8	0.342
Repetitive hand movements (Nmiss: 142)	902	10.4	1,400	15.2	**<0.001**
Repetitive pinching (Nmiss: 105)	445	5.1	626	6.8	**<0.001**
Awkward wrist postures (Nmiss: 117)	957	11.0	838	9.1	**<0.001**
Hand-transmitted vibrations our (Nmiss: 92)	443	5.1	111	1.2	**<0.001**
Cumulated exposure to biomechanical stressors (Nmiss: 378)					**<0.001**
Exposure to 1 factor	2,003	63.7	2,531	68.4	
Exposure to 2 factors	662	21.1	774	20.9	
Exposure to 3 factors	322	10.2	297	8.1	
Exposure to 4 or more factors	156	5.0	97	2.6	
**Work-related exposure to chemicals**					
Chemical exposure (at least one of the following chemicals)	1,543	17.7	531	5.7	**<0.001**
Trichlorethylene	597	6.8	128	1.4	**<0.001**
White (mineral) spirit	642	7.4	133	1.4	**<0.001**
Cellulosic diluent	333	3.8	56	0.6	**<0.001**
Pesticides	352	4.0	134	1.4	**<0.001**
Paints, varnishes	665	7.6	195	2.1	**<0.001**
Inks, dyes	203	2.3	139	1.5	**<0.001**
Cumulated exposure to chemicals					**<0.001**
Exposure to 1 chemical	824	53.4	364	68.5	
Exposure to 2 chemicals	370	24.0	107	20.2	
Exposure to 3 chemicals	209	13.6	38	7.2	
Exposure to 4 chemicals	104	6.7	17	3.2	
Exposure to 5 or more chemicals	36	2.3	5	0.9	
**Biomechanical-chemical exposure**					**<0.001**
No exposure group	4,833	55.3	5,188	55.9	
Chemical exposure group	591	6.8	186	2.0	
Biomechanical exposure group	2,357	27.0	3,566	38.4	
Co-exposure group	952	10.9	345	3.7	

CTS: carpal tunnel syndrome; Nmiss: Number of missing values.

^a^ In bold, p < 0.05.

^b^ Fisher’s test.

^c^ Technicians and associate professionals perform mostly technical and related tasks and teach at certain educational levels. Most occupations in this group require skills at the third ISCO level (education which begins at the age of 17 or 18 years and leads to an award not equivalent to a first university degree).

^d^ The blue collar worker’ category includes skilled agricultural, forestry and fishery workers (ISCO-08 group 6) and agricultural, forestry and fishery labourers (ISCO-08 group 9, elementary occupations).

184 men (2.1%, 95% CI 1.8–2.4) and 331 women (3.6%, 95% CI 3.2–3.9) free from chronic hand symptoms at baseline declared suffering from unilateral/bilateral CTS at follow-up, whether CTS required sick leave or not and/or treatment or not.

The prevalence of obesity (BMI ≥30) was relatively low (men, 9.2% and women, 9.2%), contrary to the prevalence of overweight (BMI ≥25 and <30) in men (38.0%) and, to a lesser extent, in women (20.5%). Few men (2.5%) and women (2.0%) suffered from diabetes mellitus and/or rheumatoid arthritis. A minority of men (10.0%) and women (4.0%) suffered from alcohol used disorders (Table 1).

Regarding exposure to work-related psychosocial factor, high effort–reward imbalance (ERI ratio > 1) was reported by almost half of the workers (men, 46.4%, women, 51.3%).

Regarding exposure to biomechanical wrist stressors (in the 12 month period preceding baseline), almost 4 out 10 workers (men, 37.9%, women, 42.1%) were exposed to at least one of the five biomechanical wrist stressors under study. The main biomechanical stressors were high perceived biomechanical exertion (men, 28.2%, women, 28.8%), repetitive hand movements (men, 10.4%, women, 15.2%), frequent awkward wrist postures (men, 11.0%, women, 9.1%), repetitive/forceful pinch grips (men, 5.1%, women, 6.8%), and hand-transmitted vibrations (men, 5.1%, women, 1.2%) (Table 1).

Regarding exposure to chemical exposure (for the entire occupational life), 17.7% of men and 5.7% of women were or have been exposed to at least one of the six potentially neurotoxic chemical products under study during their entire working life (Table 1). The main exposure concerned trichlorethylene (men, 6.8%, women, 1.4%) and white spirit (mineral spirit) (men, 7.4%, women, 1.4%), paints and varnishes (men, 7.6%, women, 2.1%), and pesticides (men, 4.0%, women, 1.4%). (See Table 1 for more details).

Regarding exposure to biomechanical wrist stressors and chemicals, the majority of men (55.3%) and women (55.9%) were unexposed to both biomechanical stressors and chemicals (no exposure group), 6.8% of men and 2.0% of women uniquely to chemicals (chemical exposure group) and 27.0% of men and 38.4% of women were uniquely exposed to biomechanical wrist stressors (biomechanical exposure group). Only 10.9% of men and 3.7% of women were co-exposed to chemical agents and biomechanical wrist stressors (co-exposure group) (Table 1).

As shown in S1 Table, the four groups differed according to the distribution of the occupational category with a higher proportion of blue-collar workers in the co-exposure group. The difference between the distribution of personal risk factors for CTS (age, overweight, and obesity) in the exposure groups were not clinically significant. In men, exposure to each of the five biomechanical wrist stressors differed between the co-exposure and the biomechanical exposure groups, with lower repetitive hand movements in the co-exposure group, and higher repetitive pinching, awkward wrist postures, biomechanical perceived exertion and, above all, hand-transmitted vibrations. In women, the prevalence did not differ for repetitive pinching, while exposure to repetitive hand movements, awkward wrist postures, hand-transmitted vibrations and high biomechanical perceived exertion were more frequent in co-exposed women. Exposure to some chemicals differed between the chemical exposure and co-exposure groups for both genders, including white spirit, diluents, and paints in men, and pesticides, ink and dyes in women.

### Risk models for CTS

Tables 2 and 3 show the multivariate risk models for CTS adjusted on personal and medical factors (model 1), exposure to biomechanical wrist stressors (model 2), chemicals (model 3), and exposure to biomechanical wrist stressors and chemicals (model 4).

**Table 2:** Univariate and multivariate risk models for CTS in men (N = 8,733).

				Univariate			Model 1^a^			Model 2^b^			Model 3^c^			Model 4^d^		
	N	n_CTS_	%_CTS_	OR	[95% CI]	p^e^	OR	[95% CI]	p^e^	OR	[95% CI]	p^e^	OR	[95% CI]	p^e^	OR	[95% CI]	p^e^
Age 45 or over (yrs)						**<0.001**			**0.002**			**0.001**			**0.002**			**0.001**
No	4,135	63	1.5	1			1			1			1			1		
Yes	4,598	121	2.6	1.75	[1.28–2.38]		1.67	[1.21–2.29]		1.72	[1.25–2.37]		1.64	[1.19–2.25]		1.70	[1.24–2.34]	
Diabetes mellitus and/or rheumatoid arthritis						0.749			0.431			0.400			0.441			0.429
No	8,511	180	2.1	1			1			1			1			1		
Yes	222	4	1.8	0.85	[0.31–2.31]		0.67	[0.24–1.83]		0.65	[0.24–1.78]		0.67	[0.25–1.84]		0.67	[0.24–1.83]	
Body mass index (BMI, kg/m^2^)						**<0.001**			**0.005**			**0.014**			**0.009**			**0.016**
Lean/normal < 25	4,614	73	1.6	1			1			1			1			1		
Overweight [25–30[	3,316	83	2.5	1.60	[1.16–2.19]		1.45	[1.05–2.00]		1.39	[1.01–1.92]		1.43	[1.04–1.98]		1.39	[1.01–1.92]	
Obesity ≥ 30	803	28	3.5	2.25	[1.44–3.50]		2.01	[1.28–3.15]		1.88	[1.20–2.95]		1.92	[1.22–3.01]		1.86	[1.18–2.92]	
Alcohol consumption						0.598			0.478			0.536			0.524			0.541
Abstinence	403	9	2.2	1			1			1			1			1		
Consumption without risk	2,954	56	1.9	0.85	[0.42–1.72]		0.77	[0.38–1.58]		0.85	[0.42–1.75]		0.79	[0.39–1.62]		0.86	[0.42–1.76]	
Consumption with low risk	4,505	96	2.1	0.95	[0.48–1.90]		0.95	[0.48–1.90]		1.06	[0.53–2.12]		0.97	[0.48–1.93]		1.06	[0.53–2.13]	
Alcohol use disorders	871	23	2.6	1.19	[0.54–2.59]		1.10	[0.50–2.40]		1.17	[0.53–2.57]		1.10	[0.50–2.41]		1.18	[0.54–2.58]	
Effort-reward imbalance ratio >1						0.112			0.137			0.242			0.171			0.246
No	4,682	88	1.9	1						1			1			1		
Yes	4,051	96	2.4	1.27	[0.95–1.70]		1.25	[0.93–1.68]		1.19	[0.89–1.60]		1.23	[0.92–1.65]		1.19	[0.89–1.60]	
Biomechanical exposure						**<0.001**						**<0.001**						
No	5,424	73	1.3	1						1								
Yes	3,309	111	3.4	2.54	[1.89–3.43]					2.49	[1.85–3.37]							
Chemical exposure						**<0.001**									**0.001**			
No	7,19	131	1.8	1									1					
Yes	1,543	53	3.4	1.92	[1.39–2.65]								1.77	[1.28–2.46]				
Biomechanical-chemical exposure						**<0,001**												
No exposure group	4,833	64	1.3	1												1		**<0,001**
Chemical exposure group	591	9	1.5	1.15	[0.57–2.33]											1.03	[0.51–2.09]	
Biomechanical exposure group	2,357	67	2.8	2.18	[1.54–3.08]											2.14	[1.51–3.03]	
Co-exposure group	952	44	4.6	3.61	[2.44–5.34]											3.38	[2.29–5.01]	

OR: odds-ratio; 95% CI: 95% confidence interval; BMI: body mass index.

^a^ Model 1: Including personal and medical risk factors.

^b^ Model 2: Biomechanical wrist exposure added to model 1.

^c^ Model 3: Chemical exposure added to model 1.

^d^ Model 4: Exposure to Biomechanical wrist stressors and chemical exposure including a co-exposure group added to model 1.

^e^ In bold, p < 0.05.

**Table 3:** Univariate and multivariate risk models for CTS in women (N = 9,285).

				Univariate			Model 1^a^			Model 2^b^			Model 3^c^			Model 4^d^		
	N	n_CTS_	%_CTS_	OR	[95% CI]	p^e^	OR	[95% CI]	p^e^	OR	[95% CI]	p^e^	OR	[95% CI]	p^e^	OR	[95% CI]	p^e^
Age 45 or over (yrs)						**<0.001**			**<0.001**			**0.001**			**<0.001**			**0.001**
No	4,714	133	2.8	1			1			1			1			1		
Yes	4,571	198	4.3	1.56	[1.25–1.95]		1.50	[1.20–1.89]		1.48	[1.18–1.86]		1.51	[1.20–1.89]		1.49	[1.18–1.87]	
Diabetes mellitus and/or rheumatoid arthritis						0.916			0.599			0.553			0.587			0.553
No	9,096	324	3.6	1			1			1			1			1		
Yes	189	7	3.7	1.04	[0.49–2.23]		0.81	[0.38–1.76]		0.79	[0.36–1.72]		0.81	[0.37–1.75]		0.79	[0.36–1.72]	
Body mass index (BMI, kg/m^2^)						**<0.001**			**<0.001**			**0.001**			**<0.001**			**0.001**
Lean/normal < 25	6,529	199	3.0	1			1			1			1			1		
Overweight [25–30[	1,901	79	4.2	1.38	[1.06–1.80]		1.31	[1.00–1.71]		1.28	[0.98–1.67]		1.29	[0.99–1.69]		1.26	[0.96–1.64]	
Obesity ≥ 30	855	53	6.2	2.10	[1.54–2.87]		1.97	[1.44–2.70]		1.83	[1.33–2.51]		1.94	[1.41–2.66]		1.81	[1.32–2.49]	
Alcohol consumption						0.268			0.492			0.618			0.473			0.624
Abstinence	743	33	4.4	1			1			1			1			1		
Consumption without risk	5,655	204	3.6	0.80	[0.55–1.17]		0.79	[0.54–1.16]		0.83	[0.56–1.21]		0.79	[0.54–1.15]		0.83	[0.56–1.21]	
Consumption with low risk	2,519	78	3.1	0.69	[0.45–1.04]		0.75	[0.49–1.14]		0.79	[0.52–1.20]		0.74	[0.49–1.12]		0.79	[0.52–1.20]	
Alcohol use disorders	368	16	4.3	0.98	[0.53–1.80]		0.97	[0.52–1.79]		1.01	[0.55–1.88]		0.95	[0.51–1.75]		1.00	[0.54–1.86]	
Effort-reward imbalance ratio >1						**<0.001**			**<0.001**			**<0.001**			**<0.001**			**<0.001**
No	4,523	125	2.8	1			1			1			1			1		
Yes	4,762	206	4.3	1.59	[1.27–1.99]		1.55	[1.24–1.95]		1.51	[1.21–1.90]		1.54	[1.23–1.94]		1.51	[1.20–1.89]	
Biomechanical exposure						**<0.001**						**<0.001**						
No	5,374	120	2.2	1						1								
Yes	3,911	211	5.4	2.50	[1.99–3.14]					2.36	[1.87–2.97]							
Chemical exposure						**<0.001**									**<0.001**			
No	8,754	295	3.4	1									1					
Yes	531	36	6.8	2.09	[1.46–2.98]								2.00	[1.39–2.86]				
Biomechanical-chemical exposure						**<0.001**												**<0.001**
No exposure group	5,188	116	2.2	1												1		
Chemical exposure group	186	4	2.2	0.96	[0.35–2.63]											0.93	[0.34–2.56]	
Biomechanical exposure group	3,566	179	5.0	2.31	[1.82–2.93]											2.19	[1.72–2.78]	
Co-exposure group	345	32	9.3	4.47	[2.97–6.72]											4.12	[2.73–6.21]	

OR: odds-ratio; 95% CI: 95% confidence interval; BMI: body mass index.

^a^ Model 1: Including personal and medical risk factors.

^b^ Model 2: Biomechanical wrist exposure added to model 1.

^c^ Model 3: Chemical exposure added to model 1.

^d^ Model 4: Exposure to Biomechanical wrist stressors and chemical exposure including a co-exposure group added to model 1.

^e^ In bold, p < 0.05.

Concerning personal/medical factors, age > 45 yrs was associated with CTS, with similar odds ratios for all models: ~1.7 for men and 1.5 for women. No statistical association was found with diabetes mellitus and/or rheumatoid arthritis for both genders, regardless of the model. High BMI was associated with CTS, with OR for overweight and obesity reaching ~ 1.4 and ~1.9 in men and ~ 1.3 and ~1.9 in women, respectively. Alcohol use disorders was not associated with CTS in either gender (Tables 2 and 3).

Concerning psychosocial work-related fac*tors*, high effort–reward imbalance (ERI ratio > 1) was associated with CTS in women, regardless of the model (OR ~ 1.5–1.6, p<0.001). The association in men was less strong (OR ~1.3) and not statistically significant for all models.

Concerning biomechanical work-related stressors, CTS was associated with exposure to at least one biomechanical wrist stressor for both genders (p<0.001) after adjustment for personal/medical factors (model 2, OR = 2.49 [1.85–3.37] in men and OR = 2.36 [1.87–2.97] in women).

Concerning chemical exposure, an increased risk of CTS was observed in workers exposed to chemicals after adjustment for personal/medical factors (model 3) in men (OR = 1.77 [1.28–2.46]) and women (OR = 2.00 [1.39–2.86]).

As shown in model 4, a statistically significant association (p<0.001) was found for both genders between CTS and co-exposure to biomechanical wrist stressors and chemicals, without increased risk in the chemical exposure group (without exposure to biomechanical activity). The risk of CTS approximately doubled in the biomechanical exposure group (OR = 2.14 [1.51–3.03] in men and OR = 2.19 [1.72–2.78] in women) and approximately tripled in men (OR = 3.38 [2.29–5.01]) and quadrupled in women (OR = 4.12 [2.73–6.21] in the co-exposure group compared to non-exposed workers, after adjustment for personal/medical factors and ERI ratio. The study showed a higher risk of CTS for workers co-exposed to biomechanical wrist stressors and neurotoxic chemicals in comparison to those uniquely exposed to biomechanical wrist stressors (p = 0.021 in men and p = 0.002 in women) or solely exposed to chemicals (p = 0.0001 in men and p = 0.006 in women). As shown in S2 and S3 Tables, the risk models were globally similar when the population studied was restricted to male and female lower-level white-collar workers and blue-collar workers.

## Discussion

At our knowledge, this is the first time that a study investigated prospectively the effects of co-exposure to biomechanical wrist stressors and chemicals on the risk of CTS. Our study in a large cohort of French professionally active adults found consistent associations between CTS and co-exposure to biomechanical wrist stressors and chemicals in both genders after adjustment for the personal and medical factors and ERI ratio. Importantly, the risk of CTS was approximately tripled or quadrupled in the co-exposed men and women, while it was doubled for those uniquely exposed to biomechanical wrist stressors.

### Results

The incidence of CTS in the Constances cohort was of the same order of magnitude as found in a previous French cohort conducted among the general working population of a French region [39]. The higher incidence of CTS observed in women, as the higher risk of incident CTS in older workers, are in line with previous longitudinal studies in the general [2,4,40,41] and working populations [12,39,42–44]. No statistical association was found with diabetes mellitus and/or rheumatoid arthritis, contrary to some epidemiological studies in the general population [7,9]. The study confirms previous epidemiological studies showing an association between CTS, overweight and above all obesity [6,12,44]. Adipose tissue within the carpal tunnel may gradually tighten the tunnel and increase the intracarpal pressure [45]. Obesity, as a component of the metabolic syndrome, may be associated with peripheral neuropathy, that can render the median nerve more vulnerable for compression within the carpal tunnel and against the volar ligament [6]. No increase of the risk of CTS was observed between CTS and alcohol use disorders contrary to some findings in the general population [41,46].

The study confirms the association of CTS with repetitive and/or forceful hand movements, wrist bending and hand-transmitted vibrations reported in the literature [1,10–14]. Biomechanical stressors can increase the pressure in the carpal tunnel at the wrist and lead to mechanical injury due to traction and contact stress on the median nerve [47]. The association found between CTS and effort-reward imbalance in women agrees with some epidemiological studies showing an association between CTS and work-related psychological factors, although most of them concerned the demand-control model and not the ERI model of stress at work [10,11,15,16,48].

The study showed, in both genders, a higher risk of CTS for workers co-exposed to biomechanical wrist stressors and neurotoxic chemicals in comparison to those uniquely exposed to biomechanical wrist stressors or solely exposed to chemicals, after adjustment for the main confounding factors.

Little epidemiological data is available on the impact of chemical exposure on the risk of CTS despite their potential neurotoxicity [20–22,26–29,31,36,37,49]. Chemical exposure was taken into account in a case-control study of CTS conducted in the general population of Wisconsin, but no significant association was found with CTS after adjustment for the main personal, medical and biomechanical risks [50]. A descriptive study conducted in Israeli workers exposed to prolonged low-level organophosphate exposure reported an increased risk of CTS-like symptoms [28]. Systemic peripheral neuropathies (e.g., diabetic polyneuropathy) are known to generate and diffuse subtle nerve damage, thus rendering the median nerve more vulnerable for compression within the carpal tunnel and against the volar ligament in the case of overexposure to biomechanical stressors [25,51]. According to the same reasoning, exposure to chemicals may generate subclinical changes of the median nerve [28] potentiating the mechanical stress on the nerve in cases of co-exposure to biomechanical wrist stressors. Beside impairments of the peripheral nervous system, subclinical changes of the central nervous system (CNS) may occur in workers exposed to neurotoxic chemicals decreasing the fine motor skills and sensorimotor control of finger force production [52,53]. Such CNS impairments, the most severe form being chronic solvent induced encephalopathy (CSE), may contribute to decreased motor control of finger force production and dexterity in workers performing repetitive prehensile movements, leading to lower dexterity and inefficient working performance [3]. This, in turn, may generate higher biomechanical wrist stressors and mechanical stress of the median nerve accentuating the risk of CTS in workers co-exposed to biomechanical wrist stressors. [3].

The higher risk of CTS in the co-exposure group may reflect synergic effects of biomechanical stressors and chemicals. We cannot exclude that exposure to chemicals might also be a marker for greater hand force being used at work. Given the rather crude self-reported hand-wrist exposure data, it may be that part of the effect of combined exposure is actually due to greater hand-wrist exposures. Indeed, the proportion of blue-collar workers was higher among the co-exposed and it would expect that blue-collar workers were more likely to report chemical exposure, and may have more strenuous hand use than white collar workers reporting the same level of hand use. Nevertheless, the risk models were globally similar when the population studied was restricted to lower-level white-collar workers and blue-collar workers.

#### Strengths and limitations of the study

The prospective design is one of the major strengths of the study. The Constances cohort includes a large sample of employed men and women of various age groups from different regions in France, covering a variety of occupational groups. The cohort provides a comprehensive assessment of socio-demographic, occupational and biomedical data meeting high quality standards of data collection, most of the measures having been validated in previous investigations [32]. The study sample does not represent the whole structure of professions and occupations in France as it practically excludes self-employed persons and farmers. The potential selection effects due to voluntary participation do not threaten the generalizability of results according to preliminary results of the comparison between Constances’ volunteers and a ‘control cohort’ of 400,000 nonparticipants [32], [54]. Thus, the low response rate may not affect the reported associations between co-exposure to biomechanical stressors and chemicals and CTS.

Since CTS and exposure were self-reported, we cannot exclude an inverse causality bias leading the workers most exposed to biomechanical wrist stressors and chemicals at baseline to declare more CTS at follow-up. The definition of CTS lacked specificity leading to possible misclassification bias and mild cases of CTS may have been under declared when answering the questionnaire. Due to the lack of physical examination, we cannot exclude that some cases of CTS were associated with finger flexor tendinitis or trigger finger because of overlapping symptoms. The main potential personal and medical risk factors for CTS and/or peripheral neuropathy (age, BMI, diabetes mellitus and/or arthritis, and alcohol consumption) were taken into account by the statistical models. Tobacco consumption was not retained in the models due to insufficient statistical fit of the models. Moreover associations between CTS and smoking status is uncertain and have not been assessed with adequate power in occupational studies [12,46]. Few chemicals were assessed and we cannot exclude exposure to other neurotoxic chemicals which may lead to possible misclassification of exposure. Because exposure information was self-reported, error (misclassification) in exposure estimation may have occurred due to poor recall.

#### Conclusion

This large prospective study showed higher risk of CTS in workers co-exposed to both biomechanical wrist stressors and chemicals compared to non-exposed workers, after adjustment for the main personal and medical factors. This finding supports the hypothesis of potentiating effects of chemical exposure on the risk of CTS in workers exposed to biomechanical wrist stressors. Nevertheless, future studies should confirm this result using more objective case definition of CTS (e.g., surgical cases of CTS) and chemical exposure assessment (e.g., job exposure matrix) before drawing conclusions on the synergic effects between mechanical intra-carpal stress and neurotoxic impairment of the risk of CTS.

## Supporting information

S1 Fig Study population flowchart. (TIF)

S1 File Extract from the inclusion and follow-up questionnaires. (DOCX)

S1 Table Distribution of the occupational category and personal, and potential work-related risk factors according the co-exposure groups in men (N = 8,733) and women (N = 9,285). Nmiss: Number of missing values. In bold, P < 0.05. *: Test exact de Fisher. α: Technicians and associate professionals perform mostly technical and related tasks and teach at certain educational levels. Most occupations in this group require skills at the third ISCO level (education which begins at the age of 17 or 18 years and leads to an award not equivalent to a first university degree). β: The blue collar worker’ category includes skilled agricultural, forestry and fishery workers (ISCO-08 group 6) and agricultural, forestry and fishery labourers (ISCO-08 group 9, elementary occupations).
(DOCX)

S2 Table Univariate and multivariate risk models for CTS in male (N = 1,574) low grade white collar and blue-collar workers. OR: odds-ratio; 95% CI: 95% confidence interval; BMI: body mass index. In bold, P < 0.05. Model 1: Including personal and medical risk factors; Model 2: Biomechanical wrist exposure added to model 1; Model 3: Chemical exposure added to model 1; Model 4: Exposure to Biomechanical wrist stressors and chemical exposure including a co-exposure group added to model 1.
(DOCX)

S3 Table Univariate and multivariate risk models for CTS in female (N = 2,280) low grade white collar and blue-collar workers. OR: odds-ratio; 95% CI: 95% confidence interval; BMI: body mass index. In bold, P < 0.05. Model 1: Including personal and medical risk factors; Model 2: Biomechanical wrist exposure added to model 1; Model 3: Chemical exposure added to model 1; Model 4: Exposure to Biomechanical wrist stressors and chemical exposure including a co-exposure group added to model 1.
(DOCX)

## References

[1] Palmer KT. Carpal tunnel syndrome: The role of occupational factors. Best Pract Res Clin Rheumatol. 2011;25: 15–29. 10.1016/j.berh.2011.01.014 21663847 PMC3145125

[2] Roquelaure Y, Chazelle E, Gautier L, Plaine J, Descatha A, Evanoff B, et al Time trends in incidence and prevalence of carpal tunnel syndrome over eight years according to multiple data sources: Pays de la Loire study. Scand J Work Environ Health. 2017;43: 75–85. 10.5271/sjweh.3594 27631820

[3] Li K, Evans PJ, Seitz WH, Li Z-M. Carpal tunnel syndrome impairs sustained precision pinch performance. Clin Neurophysiol. 2015;126: 194–201. 10.1016/j.clinph.2014.05.004 24877682 PMC4234695

[4] Mattioli S, Baldasseroni A, Curti S, Cooke RMT, Bena A, de Giacomi G, et al Incidence rates of in-hospital carpal tunnel syndrome in the general population and possible associations with marital status. BMC Public Health. 2008;8: 374 10.1186/1471-2458-8-374 18957090 PMC2586026

[5] Mondelli M, Curti S, Mattioli S, Aretini A, Ginanneschi F, Greco G, et al Associations Between Body Anthropometric Measures and Severity of Carpal Tunnel. Syndrome. Arch Phys Med Rehabil. 2016;97: 1456–1464. 10.1016/j.apmr.2016.03.028 27130638

[6] Shiri R, Pourmemari MH, Falah-Hassani K, Viikari-Juntura E. The effect of excess body mass on the risk of carpal tunnel syndrome: a meta-analysis of 58 studies. Obes Rev. 2015;16: 1094–1104. 10.1111/obr.12324 26395787

[7] Pourmemari MH, Shiri R. Diabetes as a risk factor for carpal tunnel syndrome: a systematic review and meta-analysis. Diabet Med. 2016;33: 10–16. 10.1111/dme.12855 26173490

[8] Shiri R. Arthritis as a risk factor for carpal tunnel syndrome: a meta-analysis. Scand J Rheumatol. 2016;45: 339–46. 10.3109/03009742.2015.1114141 27022991

[9] Mattioli S, Baldasseroni A, Bovenzi M, Curti S, Cooke RMT, Campo G, et al Risk factors for operated carpal tunnel syndrome: a multicenter population-based case-control study. BMC Public Health. 2009;9: 343 10.1186/1471-2458-9-343 19758429 PMC2761403

[10] van Rijn RM, Huisstede BMA, Koes BW, Burdorf A. Associations between work-related factors and the carpal tunnel syndrome—a systematic review. Scand J Work Environ Health. 2009;35: 19–36. 10.5271/sjweh.1306 19277433

[11] Barcenilla A, March LM, Chen JS, Sambrook PN. Carpal tunnel syndrome and its relationship to occupation: a meta-analysis. Rheumatology (Oxford). 2012;51: 250–261. 10.1093/rheumatology/ker108 21586523

[12] Harris-Adamson C, Eisen EA, Dale AM, Evanoff B, Hegmann KT, Thiese MS, et al Personal and workplace psychosocial risk factors for carpal tunnel syndrome: a pooled study cohort. Occup Environ Med. 2013;70: 529–537. 10.1136/oemed-2013-101365 23645610 PMC4508843

[13] You D, Smith AH, Rempel D. Meta-analysis: association between wrist posture and carpal tunnel syndrome among workers. Saf Health Work. 2014;5: 27–31. 10.1016/j.shaw.2014.01.003 24932417 PMC4048004

[14] Kozak A, Schedlbauer G, Wirth T, Euler U, Westermann C, Nienhaus A. Association between work-related biomechanical risk factors and the occurrence of carpal tunnel syndrome: an overview of systematic reviews and a meta-analysis of current research. BMC Musculoskelet Disord. 2015;16: 231 10.1186/s12891-015-0685-0 26323649 PMC4553935

[15] Harris-Adamson C, Eisen EA, Neophytou A, Kapellusch J, Garg A, Hegmann KT, et al Biomechanical and psychosocial exposures are independent risk factors for carpal tunnel syndrome: assessment of confounding using causal diagrams. Occup Environ Med. 2016;73: 727–734. 10.1136/oemed-2016-103634 27466616 PMC6555409

[16] Mansfield M, Thacker M, Sandford F. Psychosocial Risk Factors and the Association With Carpal Tunnel Syndrome: A Systematic Review. Hand (N Y). 2018;13: 501–508. 10.1177/1558944717736398 29078710 PMC6109903

[17] Roquelaure Y. Promoting a Shared Representation of Workers’ Activities to Improve Integrated Prevention of Work-Related Musculoskeletal Disorders. Saf Health Work. 2016;7: 171–174. 10.1016/j.shaw.2016.02.001 27340607 PMC4909852

[18] Nguyen T-H-Y, Bertin M, Bodin J, Fouquet N, Bonvallot N, Roquelaure Y. Multiple Exposures and Coexposures to Occupational Hazards Among Agricultural Workers: A Systematic Review of Observational Studies. Safety and Health at Work. 2018; 9(3): 239–248. 10.1016/j.shaw.2018.04.002 30370155 PMC6129995

[19] Vela MM, Laborda R, García AM. Neurotóxicos en el ambiente laboral: criterios de clasificación y listado provisional. Arch Prev Riesgos Labor 2003; 6(1):17–25.

[20] Simonsen L, Johnsen H, Lund SP, Matikainen E, Midtgård U, Wennberg A. Methodological approach to the evaluation of neurotoxicity data and the classification of neurotoxic chemicals. Scand J Work Environ Health. 1994;20: 1–12. 10.5271/sjweh.1435 8016593

[21] Spencer PS, Schaumburg HH, Ludolph AC, editors. Experimental and clinical neurotoxicology. 2nd ed New York: Oxford University Press; 2000.

[22] Grandjean P, Landrigan PJ. Developmental neurotoxicity of industrial chemicals. Lancet. 2006;368: 2167–2178. 10.1016/S0140-6736(06)69665-7 17174709

[23] Bertin M, Nguyen T-H-Y, Bonvallot N, Bodin J, Roquelaure Y. Occupational co-exposure to biomechanical factors and neurotoxic chemicals in a representative sample of French employees. J Occup Health. 2019 10.1002/1348-9585.12090 31747116 PMC6970399

[24] Schmid AB, Coppieters MW. The double crush syndrome revisited—a Delphi study to reveal current expert views on mechanisms underlying dual nerve disorders. Man Ther. 2011;16: 557–562. 10.1016/j.math.2011.05.005 21646036

[25] Rota E, Morelli N. Entrapment neuropathies in diabetes mellitus. World J Diabetes. 2016;7: 342–353. 10.4239/wjd.v7.i17.342 27660694 PMC5027001

[26] Bleecker ML. Vibration perception thresholds in entrapment and toxic neuropathies. J Occup Med. 1986;28: 991–994. 10.1097/00043764-198610000-00018 3772554

[27] Calvert GM, Mueller CA, Fajen JM, Chrislip DW, Russo J, Briggle T, et al Health effects associated with sulfuryl fluoride and methyl bromide exposure among structural fumigation workers. Am J Public Health. 1998;88: 1774–1780. 10.2105/ajph.88.12.1774 9842373 PMC1509053

[28] Ophir A, Karakis I, Richter ED, Abarbanel JM, Wormser U, Aschner M, et al An uncommon pattern of polyneuropathy induced by lifetime exposures to drift containing organophosphate pesticides. Neurotoxicology. 2014;45: 338–346. 10.1016/j.neuro.2014.08.004 25128617

[29] Nora DB, Gomes I, Said G, Carvalho FM, Melo A. Modifications of the sympathetic skin response in workers chronically exposed to lead. Braz J Med Biol Res. 2007;40: 81–87. 10.1590/s0100-879x2007000100011 17225000

[30] Estirado de Cabo E, Posada de la Paz M, de Andrés Copa P, Plaza Cano M del M, García de Aguinaga ML, Suarez Alvarez C, et al Carpal tunnel syndrome. A new feature in the natural history of TOS? Eur J Epidemiol. 2003;18: 983–993. 10.1023/a:1025899506009 14598929

[31] Weng C-H, Hu C-C, Yen T-H, Huang W-H. Association Between Environmental Particulate Matter and Carpal Tunnel Syndrome in Patients Undergoing Hemodialysis. Kidney Blood Press Res. 2017;42: 827–836. 10.1159/000484422 29161700

[32] Goldberg M, Carton M, Descatha A, Leclerc A, Roquelaure Y, Santin G, et al CONSTANCES: a general prospective population-based cohort for occupational and environmental epidemiology: cohort profile. Occup Environ Med. 2017;74: 66–71. 10.1136/oemed-2016-103678 27884936 PMC5241503

[33] Bush K, Kivlahan DR, McDonell MB, Fihn SD, Bradley KA, for the Ambulatory Care Quality Improvement Project (ACQUIP). The audit alcohol consumption questions (audit-c): An effective brief screening test for problem drinking. Arch Intern Med. 1998;158: 1789–1795. 10.1001/archinte.158.16.1789 9738608

[34] Siegrist J, Wahrendorf M, Goldberg M, Zins M, Hoven H. Is effort-reward imbalance at work associated with different domains of health functioning? Baseline results from the French CONSTANCES study. Int Arch Occup Environ Health. 2019;92: 467–480. 10.1007/s00420-018-1374-8 30406331

[35] Sluiter JK, Rest KM, Frings-Dresen MH. Criteria document for evaluating the work-relatedness of upper-extremity musculoskeletal disorders. Scand J Work Environ Health. 2001;27 Suppl 1: 1–102. 10.5271/sjweh.637 11401243

[36] LoPachin RM, Gavin T. Toxic Neuropathies: Mechanistic Insights Based On A Chemical Perspective. Neurosci Lett. 2015;596: 78–83. 10.1016/j.neulet.2014.08.054 25218479 PMC4362956

[37] Richardson JR, Fitsanakis V, Westerink RHS, Kanthasamy AG. Neurotoxicity of pesticides. Acta Neuropathol. 2019;138: 343–362. 10.1007/s00401-019-02033-9 31197504 PMC6826260

[38] Silverstein B, Fan ZJ, Smith CK, Bao S, Howard N, Spielholz P, et al Gender adjustment or stratification in discerning upper extremity musculoskeletal disorder risk? Scand J Work Environ Health. 2009;35: 113–126. 10.5271/sjweh.1309 19294319

[39] Petit A, Ha C, Bodin J, Rigouin P, Descatha A, Brunet R, et al Risk factors for carpal tunnel syndrome related to the work organization: A prospective surveillance study in a large working population. Applied Ergonomics. 2015;47: 1–10. 10.1016/j.apergo.2014.08.007 25479968

[40] Mondelli M, Giannini F, Giacchi M. Carpal tunnel syndrome incidence in a general population. Neurology. 2002;58: 289–294. 10.1212/wnl.58.2.289 11805259

[41] Bland J, Rudolfer S. Clinical surveillance of carpal tunnel syndrome in two areas of the United Kingdom, 1991–2001. J Neurol Neurosurg Psychiatry. 2003;74: 1674–1679. 10.1136/jnnp.74.12.1674 14638888 PMC1757436

[42] da Costa BR, Vieira ER. Risk factors for work-related musculoskeletal disorders: A systematic review of recent longitudinal studies. Am J Ind Med. 2010;53: 285–323. 10.1002/ajim.20750 19753591

[43] Dale AM, Harris-Adamson C, Rempel D, Gerr F, Hegmann K, Silverstein B, et al Prevalence and incidence of carpal tunnel syndrome in US working populations: pooled analysis of six prospective studies. Scand J Work Environ Health. 2013;39: 495–505. 10.5271/sjweh.3351 23423472 PMC4042862

[44] Violante FS, Farioli A, Graziosi F, Marinelli F, Curti S, Armstrong TJ, et al Carpal tunnel syndrome and manual work: the OCTOPUS cohort, results of a ten-year longitudinal study. Scand J Work Environ Health. 2016;42: 280–290. 10.5271/sjweh.3566 27159901

[45] Bland JDP. The relationship of obesity, age, and carpal tunnel syndrome: more complex than was thought? Muscle Nerve. 2005;32: 527–532. 10.1002/mus.20408 16025527

[46] Pourmemari M-H, Viikari-Juntura E, Shiri R. Smoking and carpal tunnel syndrome: a meta-analysis. Muscle Nerve. 2014;49: 345–350. 10.1002/mus.23922 23761223

[47] Viikari-Juntura E, Silverstein B. Role of physical load factors in carpal tunnel syndrome. Scand J Work Environ Health. 1999;25: 163–185. 10.5271/sjweh.423 10450768

[48] Koch P, Schablon A, Latza U, Nienhaus A. Musculoskeletal pain and effort-reward imbalance—a systematic review. BMC Public Health. 2014;14: 37 10.1186/1471-2458-14-37 24428955 PMC3898401

[49] Park SK, Kong KA, Cha ES, Lee YJ, Lee GT, Lee WJ. Occupational exposure to pesticides and nerve conduction studies among Korean farmers. Arch Environ Occup Health. 2012;67: 78–83. 10.1080/19338244.2011.573022 22524647

[50] Nordstrom DL, Vierkant RA, DeStefano F, Layde PM. Risk factors for carpal tunnel syndrome in a general population. Occup Environ Med. 1997;54: 734–740. 10.1136/oem.54.10.734 9404321 PMC1128928

[51] Horinouchi S, Deguchi T, Arimura K, Arimura A, Dochi Y, Uto T, et al Median neuropathy at the wrist as an early manifestation of diabetic neuropathy. J Diabetes Investig. 2014;5: 709–713. 10.1111/jdi.12211 25422772 PMC4234235

[52] van Valen E, Wekking E, van der Laan G, Sprangers M, van Dijk F. The course of chronic solvent induced encephalopathy: a systematic review. Neurotoxicology. 2009;30: 1172–1186. 10.1016/j.neuro.2009.06.002 19538991

[53] Kim E-A, Kang S-K. Occupational neurological disorders in Korea. J Korean Med Sci. 2010;25: S26–35. 10.3346/jkms.2010.25.S.S26 21258587 PMC3023358

[54] Rothman KJ, Gallacher JEJ, Hatch EE. Why representativeness should be avoided. Int J Epidemiol. 2013;42: 1012–1014. 10.1093/ije/dys223 24062287 PMC3888189

