# Supplementary figures and images for: Carpal tunnel syndrome and exposure to work-related biomechanical stressors and chemicals: Findings from the Constances cohort
Source: PLoS One. 2020 Jun 25;15(6):e0235051. doi: 10.1371/journal.pone.0235051 (PMC7316232; doi:10.1371/journal.pone.0235051)

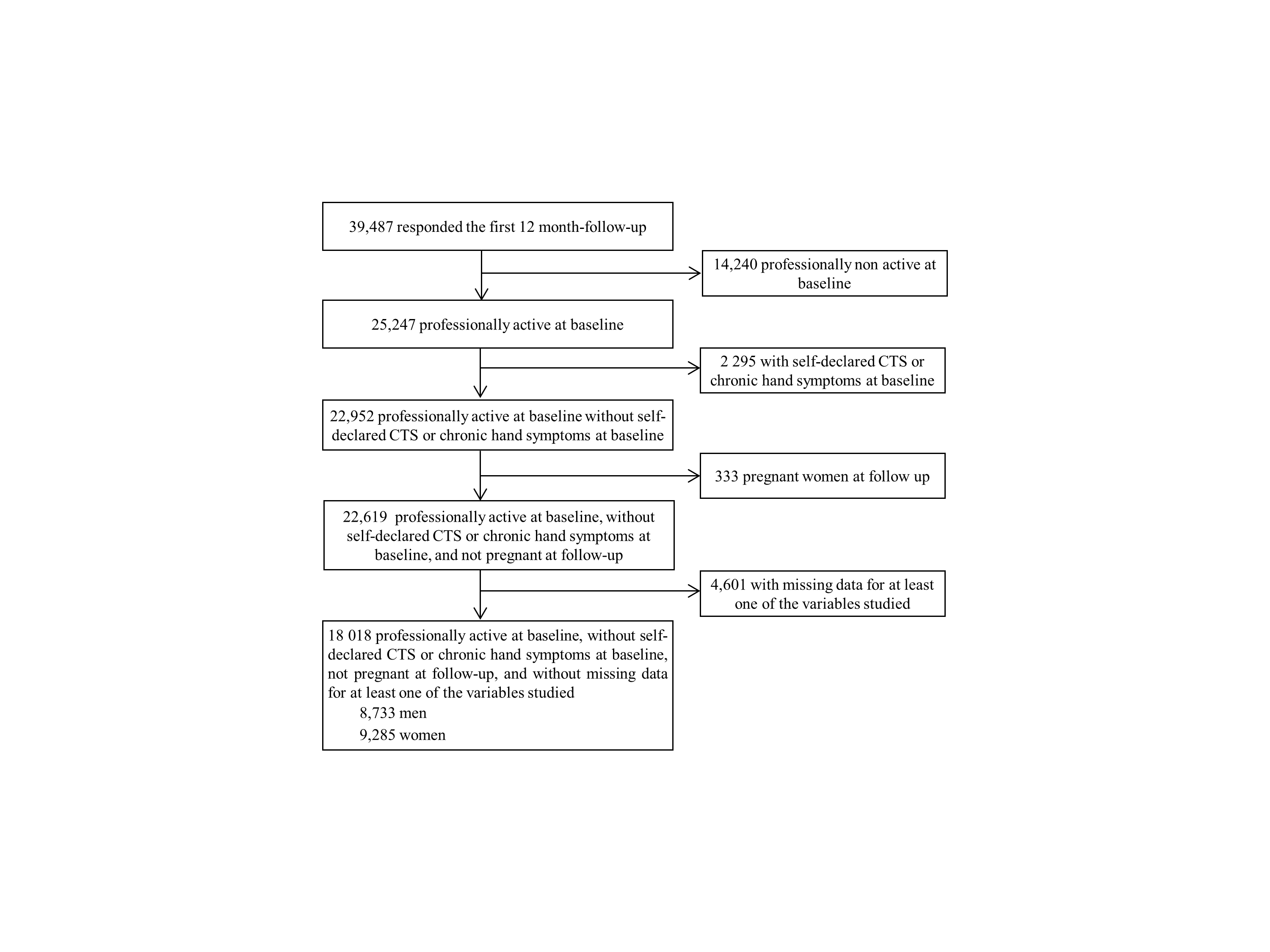

Supplement: S1 Fig — (TIF) [file pone.0235051.s001.TIF]
