# Supplementary material for: Carpal tunnel syndrome and exposure to work-related biomechanical stressors and chemicals: Findings from the Constances cohort
Source: PLoS One. 2020 Jun 25;15(6):e0235051. doi: 10.1371/journal.pone.0235051 (PMC7316232; doi:10.1371/journal.pone.0235051)
Supplement: S1 File — (DOCX) [file pone.0235051.s002.docx]

**S1 Questionnaire. Extract from the inclusion and follow-up questionnaires**

***Lifestyles and health (Self-questionnaire to be filled in at home)***

**Alcohol**

Have you ever consumed alcoholic beverages (wine, apéritif, eider, beer, etc.)?

Yes

No

How frequently do you drink alcohol?

Never

Once per month or less

2 to 4 limes per month

2 to 3 limes per week

At least 4 limes per week

How many glasses of alcohol do you drink on a typical drinking day?

1 or 2

3 or 4

5 or 6

7 to 9

10 or more

How often do you drink six or more glasses in one session?

Never

Less than once per month

Once per month

Once per week

Every day, or nearly

**Life at work**

|  | Disagree strongly | Disagree | Agree | Fully agree |
| --- | --- | --- | --- | --- |
| I am currently rushed for time due to a heavy workload | 🞎 | 🞎 | 🞎 | 🞎 |
| I am frequently interrupted and disturbed at work | 🞎 | 🞎 | 🞎 | 🞎 |
| Over the past few years, my work has become increasingly demanding | 🞎 | 🞎 | 🞎 | 🞎 |
| I get the respect I deserve from my superiors | 🞎 | 🞎 | 🞎 | 🞎 |
| My promotion prospects are poor | 🞎 | 🞎 | 🞎 | 🞎 |
| I am currently experiencing, or expect to experience an undesirable change in my working conditions | 🞎 | 🞎 | 🞎 | 🞎 |
| My job safety is at risk | 🞎 | 🞎 | 🞎 | 🞎 |
| Considering all my efforts, I get the respect and esteem I deserve at my work. | 🞎 | 🞎 | 🞎 | 🞎 |
| Considering all my efforts, my promotion prospects are satisfactory | 🞎 | 🞎 | 🞎 | 🞎 |
| Considering all my efforts, my salary is satisfactory | 🞎 | 🞎 | 🞎 | 🞎 |
| I am frequently pushed for time at work | 🞎 | 🞎 | 🞎 | 🞎 |
| I start to think of work-related problems as soon as I wake up in the morning | 🞎 | 🞎 | 🞎 | 🞎 |
| When I get home, I can easily relax and forget about my work | 🞎 | 🞎 | 🞎 | 🞎 |
| My friends and family say that I give up too much for my job | 🞎 | 🞎 | 🞎 | 🞎 |
| I am still preoccupied by work when I go to bed | 🞎 | 🞎 | 🞎 | 🞎 |
| If I put something back that I should have done that day, I have trouble getting to sleep | 🞎 | 🞎 | 🞎 | 🞎 |

***Medical questionnaire (Questionnaire managed by the physician)***

**Personal medical history**

Diabetes

***Paraclinical examination data collection form (Questionnaire administered by the health clinic)***

**Biometry**

Height

Weight

***Occupational exposures (Questionnaire managed by the health clinic)***

**Complete professional career**

**Exposure to chemicals**

During your professional life, have you been (or are you currently) in contact with the following nuisances?

**Solvents, diluents, degreasing agents (excluding soap) or disinfectants (for cleaning equipment or your hands), such as:**

Trichlorethylene

Yes If yes, from what year to what year: [from YYYY to YYYY] X 3

No

Don't know

White spirit

Yes If yes, from what year to what year: [from YYYY to YYYY] X 3

No

Don't know

Cellulose diluent

Yes If yes, from what year to what year: [from YYYY to YYYY] X 3

No

Don't know

**Other nuisances**

Pesticides (weed killers, insecticides, fungicides)

Yes If yes, from what year to what year: [from YYYY to YYYY] X 3

No

Don't know

Paint, varnish

Yes If yes, from what year to what year: [from YYYY to YYYY] X 3

No

Don't know

Ink, stains

Yes If yes, from what year to what year: [from YYYY to YYYY] X 3

No

Don't know

**Current job**

**Postural stress**

How would you rate the intensity of physical effort during a typical day at work? ***Tick the figure matching your choice on the scale from*** *6 to 20 below, ranging from “No exertion at all” to “Maximal exertion”*

6 No exertion at all

7 Extremely light

8

9 Very light

10

11 Light

12

13 Somewhat hard

14

15 Hard

16

17 Very hard

18

19 Extremely hard

20 Maximal exertion

|  | Never or almost never | Rarely (less than 2 hours a day) | Often (2 to 4 hours a day) | Most of the time (more than 4 hours a day) |
| --- | --- | --- | --- | --- |
| During a typical working day, do you need to repeat the same actions more than 2 to 4 times per minute? | 🞎 | 🞎 | 🞎 | 🞎 |

|  | Never or almost never | Rarely (less than 2 hours a day) | Often (2 to 4 hours a day) | Most of the time (more than 4 hours a day) |
| --- | --- | --- | --- | --- |
| During a typical working day do you use vibrating tools, or are you required to place your hand(s) on vibrating machines? | 🞎 | 🞎 | 🞎 | 🞎 |

During a typical working day, during how much time do you have to adopt the following positions?

|  |  | Never or almost never | Rarely (less than 2 hours a day) | Often (2 to 4 hours a day) | Most of the time (more than 4 hours a day) |
| --- | --- | --- | --- | --- | --- |
| Twist the wrist? |  | 🞎 | 🞎 | 🞎 | 🞎 |
| Press or firmly grip objects or parts between thumb and index finger? |  | 🞎 | 🞎 | 🞎 | 🞎 |

***Follow-up questionnaire (Self-questionnaire to be filled in at home)***

Below is a list of health problems. Indicate those from which you suffer or have suffered over the past 12 months (whether or not accompanied by occupational leave, whether or not accompanied by treatment).

Bone and joint disease

- Sciatica
- Lower back pains
- Neck pain
- Joint or muscle pain:
  - Shoulder
  - Elbow, hand
  - Knee, hip
- Carpal tunnel syndrome
- Osteoporosis
- Rheumatoid arthritis, ankylosing spondylitis or polymyalgia rheumatica (PR)
