# Supplementary material for: Carpal tunnel syndrome and exposure to work-related biomechanical stressors and chemicals: Findings from the Constances cohort
Source: PLoS One. 2020 Jun 25;15(6):e0235051. doi: 10.1371/journal.pone.0235051 (PMC7316232; doi:10.1371/journal.pone.0235051)
Supplement: S1 Table — Nmiss: Number of missing values. In bold, P < 0.05. *: Test exact de Fisher. α: Technicians and associate professionals perform mostly technical and related tasks and teach at certain educational levels. Most occupations in this group require skills at the third ISCO level (education which begins at the age of 17 or 18 years and leads to an award not equivalent to a first university degree). β: The blue collar worker’ category includes skilled agricultural, forestry and fishery workers (ISCO-08 group 6) and agricultural, forestry and fishery labourers (ISCO-08 group 9, elementary occupations). (DOCX) [file pone.0235051.s003.docx]

**S1 Table. Distribution of the occupational category and personal, and potential work-related risk factors according the co-exposure groups in men (N=8,733) and women (N=9,285).**

|  | **Men (N=8,733)** | | | | | | | | | **Women (N=9,285)** | | | | | | | | |
| --- | --- | --- | --- | --- | --- | --- | --- | --- | --- | --- | --- | --- | --- | --- | --- | --- | --- | --- |
|  | **No exposure group** | | **Chemical exposure group** | | **Biomechanical exposure group** | | **Co-exposure group** | | **p**^a^ | **No exposure group** | | **Chemical exposure group** | | **Biomechanical exposure group** | | **Co-exposure group** | | **p**^a^ |
|  | **N** | **%** | **N** | **%** | **N** | **%** | **N** | **%** |  | **N** | **%** | **N** | **%** | **N** | **%** | **N** | **%** |  |
| **Age 45 or more (yrs)** | 2,540 | 52.6 | 365 | 61.8 | 1,163 | 49.3 | 530 | 55.7 | **<0.001** | 2,483 | 47.9 | 89 | 47.9 | 1,825 | 51.2 | 174 | 50.4 | **0.022** |
| **Diabetes and/or rheumatoid arthritis** | 109 | 2.3 | 20 | 3.4 | 71 | 3.0 | 22 | 2.3 | 0.133 | 98 | 1.9 | 5 | 2.7 | 78 | 2.2 | 8 | 2.3 | 0.680 |
| **Body mass index** |  |  |  |  |  |  |  |  | **<0.001** |  |  |  |  |  |  |  |  | **<0.001** |
| Underweight/normal (< 25 kg/m²) | 2,697 | 55.8 | 275 | 46.5 | 1189 | 50.5 | 453 | 47.6 |  | 3,770 | 72.7 | 128 | 68.8 | 2,425 | 68.0 | 206 | 59.7 |  |
| Overweight [25-30 kg/m²[ | 1,760 | 36.4 | 234 | 39.6 | 936 | 39.7 | 386 | 40.5 |  | 1,024 | 19.7 | 40 | 21.5 | 741 | 20.8 | 96 | 27.8 |  |
| Obesity (≥ 30 kg/m²) | 376 | 7.8 | 82 | 13.9 | 232 | 9.8 | 113 | 11.9 |  | 394 | 7.6 | 18 | 9.7 | 400 | 11.2 | 43 | 12.5 |  |
| **Alcohol consumption** |  |  |  |  |  |  |  |  | **<0.001** |  |  |  |  |  |  |  |  | **0.002** |
| Abstinence | 179 | 3.7 | 25 | 4.2 | 141 | 6.0 | 58 | 6.1 |  | 380 | 7.3 | 6 | 3.2 | 327 | 9.2 | 30 | 8.7 |  |
| Consumption without risk | 1,669 | 34.5 | 190 | 32.2 | 779 | 33.0 | 316 | 33.2 |  | 3,133 | 60.4 | 119 | 64.0 | 2,202 | 61.8 | 201 | 58.3 |  |
| Consumption with low risk | 2,543 | 52.6 | 308 | 52.1 | 1,180 | 50.1 | 474 | 49.8 |  | 1,472 | 28.4 | 52 | 28.0 | 900 | 25.2 | 95 | 27.5 |  |
| Alcohol use disorders | 442 | 9.2 | 68 | 11.5 | 257 | 10.9 | 104 | 10.9 |  | 203 | 3.9 | 9 | 4.8 | 137 | 3.8 | 19 | 5.5 |  |
| **Occupational category** (Nmiss: 2 459) |  |  |  |  |  |  |  |  | **<0.001**^b^ |  |  |  |  |  |  |  |  | **<0.001**^b^ |
| 1 - Farmers | 0 | 0.0 | 0 | 0.0 | 0 | 0.0 | 2 | 0.3 |  | 0 | 0.0 | 0 | 0.0 | 0 | 0.0 | 2 | 0.7 |  |
| 2 – Craftsmen. salesmen and managers | 47 | 1.3 | 11 | 2.7 | 36 | 1.9 | 15 | 2.2 |  | 26 | 0.6 | 2 | 1.4 | 17 | 0.6 | 4 | 1.5 |  |
| 3 – Professionals | 2,434 | 67.1 | 173 | 42.0 | 543 | 29.2 | 74 | 10.6 |  | 1,662 | 40.4 | 50 | 35.5 | 596 | 19.5 | 32 | 11.6 |  |
| 4 - Technicians and associate professionals^c^ | 838 | 23.1 | 158 | 38.3 | 539 | 28.9 | 156 | 22.4 |  | 1,561 | 38.0 | 55 | 39.0 | 1,216 | 39.7 | 82 | 29.7 |  |
| 5 – Low grade white collar workers | 213 | 5.9 | 33 | 8.0 | 251 | 13.5 | 88 | 12.6 |  | 837 | 20.4 | 32 | 22.7 | 1,119 | 36.6 | 109 | 39.5 |  |
| 6 – Blue collar workers^d^ | 96 | 2.6 | 37 | 9.0 | 493 | 26.5 | 363 | 52.0 |  | 24 | 0.6 | 2 | 1.4 | 110 | 3.6 | 47 | 17.0 |  |
| **Biomechanical exposure (at least one of the following factor)** |  |  |  |  |  |  |  |  |  |  |  |  |  |  |  |  |  |  |
| High physical perceived exertion (RPE ≥12) (Nmiss: 65) |  |  |  |  | 1,659 | 70.9 | 791 | 84.6 | **<0.001** |  |  |  |  | 2403 | 67.9 | 262 | 77.5 | **<0.001** |
| Repetitive hand movements (Nmiss: 142) |  |  |  |  | 680 | 29.3 | 222 | 23.8 | **0.001** |  |  |  |  | 1256 | 36.0 | 144 | 43.1 | **0.010** |
| Repetitive pinching (Nmiss: 105) |  |  |  |  | 296 | 12.7 | 149 | 15.9 | **0.018** |  |  |  |  | 562 | 16.0 | 64 | 18.7 | 0.197 |
| Awkward wrist postures (Nmiss: 117) |  |  |  |  | 602 | 26.0 | 355 | 37.7 | **<0.001** |  |  |  |  | 728 | 20.8 | 110 | 32.1 | **<0.001** |
| Hand-transmitted vibrations our (Nmiss: 92) |  |  |  |  | 198 | 8.5 | 245 | 26.0 | **<0.001** |  |  |  |  | 79 | 2.2 | 32 | 9.4 | **<0.001** |
| **Chemical exposure (at least one of the following chemicals)** |  |  |  |  |  |  |  |  |  |  |  |  |  |  |  |  |  |  |
| Trichlorethylene |  |  | 238 | 40.3 |  |  | 359 | 37.7 | 0.315 |  |  | 51 | 27.4 |  |  | 77 | 22.3 | 0.190 |
| White (mineral) spirit |  |  | 226 | 38.2 |  |  | 416 | 43.7 | **0.035** |  |  | 45 | 24.2 |  |  | 88 | 25.5 | 0.739 |
| Cellulosic diluent |  |  | 104 | 17.6 |  |  | 229 | 24.1 | **0.003** |  |  | 23 | 12.4 |  |  | 33 | 9.6 | 0.316 |
| Pesticides |  |  | 121 | 20.5 |  |  | 231 | 24.3 | 0.085 |  |  | 37 | 19.9 |  |  | 97 | 28.1 | **0.037** |
| Paints, varnishes |  |  | 212 | 35.9 |  |  | 453 | 47.6 | **<0.001** |  |  | 77 | 41.4 |  |  | 118 | 34.2 | 0.101 |
| Inks, dyes |  |  | 71 | 12.0 |  |  | 132 | 13.9 | 0.295 |  |  | 38 | 20.4 |  |  | 101 | 29.3 | **0.027** |
| Nmiss: Number of missing values.  ^a^ In bold, P < 0.05.  ^b^ Fisher’s test.  ^c^ Technicians and associate professionals perform mostly technical and related tasks and teach at certain educational levels. Most occupations in this group require skills at the third ISCO level (education which begins at the age of 17 or 18 years and leads to an award not equivalent to a first university degree).  ^d^ The blue collar worker’ category includes skilled agricultural, forestry and fishery workers (ISCO-08 group 6) and agricultural, forestry and fishery labourers (ISCO-08 group 9, elementary occupations). | | | | | | | | | | | | | | | | | | |
