# Supplementary material for: Carpal tunnel syndrome and exposure to work-related biomechanical stressors and chemicals: Findings from the Constances cohort
Source: PLoS One. 2020 Jun 25;15(6):e0235051. doi: 10.1371/journal.pone.0235051 (PMC7316232; doi:10.1371/journal.pone.0235051)
Supplement: S2 Table — OR: odds-ratio; 95% CI: 95% confidence interval; BMI: body mass index. In bold, P < 0.05. Model 1: Including personal and medical risk factors; Model 2: Biomechanical wrist exposure added to model 1; Model 3: Chemical exposure added to model 1; Model 4: Exposure to Biomechanical wrist stressors and chemical exposure including a co-exposure group added to model 1. (DOCX) [file pone.0235051.s004.docx]

**S2 Table. Univariate and multivariate risk models for CTS in male low grade white collar and blue-collar workers (N=1,574).**

|  |  |  |  | Univariate | | | Model 1^a^ | | | Model 2^b^ | | | Model 3^c^ | | | Model 4^d^ | | |
| --- | --- | --- | --- | --- | --- | --- | --- | --- | --- | --- | --- | --- | --- | --- | --- | --- | --- | --- |
|  | N | n_CTS_ | %_CTS_ | OR | [95% CI] | p^e^ | OR | [95% CI] | p^e^ | OR | [95% CI] | p^e^ | OR | [95% CI] | p^e^ | OR | [95% CI] | p^e^ |
| Age 45 or more (yrs) |  |  |  |  |  | 0.161 |  |  | 0.133 |  |  | 0.100 |  |  | 0.157 |  |  | 0.114 |
| No | 748 | 21 | 2.8 | 1 |  |  | 1 |  |  | 1 |  |  | 1 |  |  | 1 |  |  |
| Yes | 826 | 34 | 4.1 | 1.49 | [0.85-2.58] |  | 1.55 | [0.88-2.74] |  | 1.61 | [0.91-2.86] |  | 1.51 | [0.85-2.67] |  | 1.59 | [0.90-2.82] |  |
| Diabetes mellitus and/or rheumatoid arthritis |  |  |  |  |  | 0.953 |  |  | 0.983 |  |  | 0.967 |  |  | 0.946 |  |  | 0.987 |
| No | 1,519 | 53 | 3.5 | 1 |  |  | 1 |  |  | 1 |  |  | 1 |  |  | 1 |  |  |
| Yes | 55 | 2 | 3.6 | 1.04 | [0.25-4.40] |  | 1.02 | [0.24-4.34] |  | 0.97 | [0.23-4.15] |  | 1.05 | [0.25-4.50] |  | 0.99 | [0.23-4.24] |  |
| Body mass index |  |  |  |  |  | 0.920 |  |  | 0.767 |  |  | 0.757 |  |  | 0.754 |  |  | 0.738 |
| Underweight/normal (< 25 kg/m²) | 738 | 27 | 3.7 | 1 |  |  | 1 |  |  | 1 |  |  | 1 |  |  | 1 |  |  |
| Overweight [25-30 kg/m²[ | 641 | 22 | 3.4 | 0.94 | [0.53-1.66] |  | 0.87 | [0.49-1.55] |  | 0.86 | [0.48-1.53] |  | 0.87 | [0.49-1.56] |  | 0.85 | [0.48-1.53] |  |
| Obesity (≥ 30 kg/m²) | 195 | 6 | 3.1 | 0.84 | [0.34-2.05] |  | 0.73 | [0.29-1.82] |  | 0.73 | [0.29-1.83] |  | 0.72 | [0.29-1.80] |  | 0.72 | [0.29-1.79] |  |
| Alcohol consumption | |  |  |  |  | 0.934 |  |  | 0.954 |  |  | 0.973 |  |  | 0.955 |  |  | 0.974 |
| Abstinence | 127 | 4 | 3.1 | 1 |  |  | 1 |  |  | 1 |  |  | 1 |  |  | 1 |  |  |
| Consumption without risk | 499 | 17 | 3.4 | 1.08 | [0.36-3.28] |  | 1.03 | [0.34-3.12] |  | 1.09 | [0.36-3.32] |  | 1.02 | [0.33-3.10] |  | 1.07 | [0.35-3.27] |  |
| Consumption with low risk | 762 | 26 | 3.4 | 1.09 | [0.37-3.17] |  | 1.09 | [0.37-3.18] |  | 1.11 | [0.38-3.26] |  | 1.07 | [0.37-3.13] |  | 1.09 | [0.37-3.20] |  |
| Alcohol use disorders | 186 | 8 | 4.3 | 1.38 | [0.41-4.69] |  | 1.30 | [0.38-4.45] |  | 1.30 | [0.38-4.44] |  | 1.29 | [0.38-4.42] |  | 1.28 | [0.37-4.39] |  |
| Effort-reward imbalance ratio >1 | |  |  |  |  | 0.340 |  |  | 0.326 |  |  | 0.465 |  |  | 0.366 |  |  | 0.481 |
| No | 900 | 28 | 3.1 | 1 |  |  | 1 |  |  | 1 |  |  | 1 |  |  | 1 |  |  |
| Yes | 674 | 27 | 4.0 | 1.30 | [0.76-2.23] |  | 1.31 | [0.76-2.25] |  | 1.22 | [0.71-2.11] |  | 1.28 | [0.75-2.21] |  | 1.22 | [0.71-2.10] |  |
| Biomechanical wrist exposure |  |  |  |  |  | **0.025** |  |  |  |  |  | **0.025** |  |  |  |  |  |  |
| No | 379 | 6 | 1.6 | 1 |  |  |  |  |  | 1 |  |  |  |  |  |  |  |  |
| Yes | 1,195 | 49 | 4.1 | 2.66 | [1.13-6.25] |  |  |  |  | 2.68 | [1.13-6.35] |  |  |  |  |  |  |  |
| Chemical exposure |  |  |  |  |  | 0.164 |  |  |  |  |  |  |  |  | 0.213 |  |  |  |
| No | 1,053 | 32 | 3.0 | 1 |  |  |  |  |  |  |  |  | 1 |  |  |  |  |  |
| Yes | 521 | 23 | 4.4 | 1.47 | [0.85-2.54] |  |  |  |  |  |  |  | 1.42 | [0.82-2.46] |  |  |  |  |
| Biomechanical-chemical co-exposure |  |  |  |  |  | 0.119 |  |  |  |  |  |  |  |  |  |  |  | 0.131 |
| No exposure group | 309 | 4 | 1.3 | 1 |  |  |  |  |  |  |  |  |  |  |  | 1 |  |  |
| Chemical exposure group | 744 | 28 | 3.8 | 2.24 | [0.40-12.49] |  |  |  |  |  |  |  |  |  |  | 2.28 | [0.41-12.75] |  |
| Biomechanical exposure group | 70 | 2 | 2.9 | 2.98 | [1.04-8.57] |  |  |  |  |  |  |  |  |  |  | 3.08 | [1.07-8.90] |  |
| Co-exposure group | 451 | 21 | 4.7 | 3.72 | [1.27-10.96] |  |  |  |  |  |  |  |  |  |  | 3.65 | [1.23-10.79] |  |
| OR: odds-ratio; 95% CI: 95% confidence interval; BMI: body mass index. | | | | | | | | | | | | | | | | | | |
| ^a^ Model 1: Including personal and medical risk factors. | | | | | | | | | | | | | | | | | | |
| ^b^ Model 2: Biomechanical wrist exposure added to model 1. | | | | | | | | | | | | | | | | | | |
| ^c^ Model 3: Chemical exposure added to model 1. | | | | | | | | | | | | | | | | | | |
| ^d^ Model 4: Exposure to Biomechanical wrist stressors and chemical exposure including a co-exposure group added to model 1. | | | | | | | | | | | | | | | | | | |
| ^e^ In bold, p < 0.05. | | | | | | | | | | | | | | | | | | |
