# Supplementary material for: Carpal tunnel syndrome and exposure to work-related biomechanical stressors and chemicals: Findings from the Constances cohort
Source: PLoS One. 2020 Jun 25;15(6):e0235051. doi: 10.1371/journal.pone.0235051 (PMC7316232; doi:10.1371/journal.pone.0235051)
Supplement: S3 Table — OR: odds-ratio; 95% CI: 95% confidence interval; BMI: body mass index. In bold, P < 0.05. Model 1: Including personal and medical risk factors; Model 2: Biomechanical wrist exposure added to model 1; Model 3: Chemical exposure added to model 1; Model 4: Exposure to Biomechanical wrist stressors and chemical exposure including a co-exposure group added to model 1. (DOCX) [file pone.0235051.s005.docx]

**S3 Table. Univariate and multivariate risk models for CTS in female low grade white collar and blue-collar workers(N=2,280).**

|  |  |  |  | Univariate | | | Model 1^a^ | | | Model 2^b^ | | | Model 3^c^ | | | Model 4^d^ | | |
| --- | --- | --- | --- | --- | --- | --- | --- | --- | --- | --- | --- | --- | --- | --- | --- | --- | --- | --- |
|  | N | n_CTS_ | %_CTS_ | OR | [95% CI] | p^e^ | OR | [95% CI] | p^e^ | OR | [95% CI] | p^e^ | OR | [95% CI] | p^e^ | OR | [95% CI] | p^e^ |
| Age 45 or more (yrs) |  |  |  |  |  | 0.137 |  |  | 0.275 |  |  | 0.300 |  |  | 0.294 |  |  | 0.323 |
| No | 1,082 | 53 | 4.9 | 1 |  |  | 1 |  |  | 1 |  |  | 1 |  |  | 1 |  |  |
| Yes | 1,198 | 76 | 6.3 | 1.32 | [0.92-1.89] |  | 1.23 | [0.85-1.77] |  | 1.22 | [0.84-1.76] |  | 1.22 | [0.84-1.76] |  | 1.21 | [0.83-1.75] |  |
| Diabetes mellitus and/or rheumatoid arthritis |  |  |  |  |  | 0.124 |  |  | 0.332 |  |  | 0.256 |  |  | 0.330 |  |  | 0.255 |
| No | 2,222 | 123 | 5.5 | 1 |  |  | 1 |  |  | 1 |  |  | 1 |  |  | 1 |  |  |
| Yes | 58 | 6 | 10.3 | 1.97 | [0.83-4.67] |  | 1.55 | [0.64-3.76] |  | 1.68 | [0.69-4.11] |  | 1.56 | [0.64-3.78] |  | 1.68 | [0.69-4.13] |  |
| Body mass index |  |  |  |  |  | **0.025** |  |  | **0.049** |  |  | 0.115 |  |  | 0.067 |  |  | 0.143 |
| Underweight/normal (< 25 kg/m²) | 1,43 | 69 | 4.8 | 1 |  |  | 1 |  |  | 1 |  |  | 1 |  |  | 1 |  |  |
| Overweight [25-30 kg/m²[ | 527 | 32 | 6.1 | 1.28 | [0.83-1.96] |  | 1.25 | [0.81-1.93] |  | 1.21 |  |  | 1.22 | [0.79-1.89] |  | 1.19 | [0.77-1.84] |  |
| Obesity (≥ 30 kg/m²) | 323 | 28 | 8.7 | 1.87 | [1.19-2.96] |  | 1.79 | [1.12-2.86] |  | 1.64 | [1.03-2.63] |  | 1.74 | [1.09-2.79] |  | 1.61 | [1.00-2.57] |  |
| Alcohol consumption |  |  |  |  |  | 0.618 |  |  | 0.697 |  |  | 0.741 |  |  | 0.654 |  |  | 0.693 |
| Abstinence | 280 | 16 | 5.7 | 1 |  |  | 1 |  |  | 1 |  |  | 1 |  |  | 1 |  |  |
| Consumption without risk | 1,373 | 84 | 6.1 | 1.08 | [0.62-1.87] |  | 1.16 | [0.66-2.03] |  | 1.14 | [0.65-2.01] |  | 1.15 | [0.66-2.02] |  | 1.14 | [0.65-2.00] |  |
| Consumption with low risk | 540 | 25 | 4.6 | 0.80 | [0.42-1.53] |  | 0.89 | [0.46-1.71] |  | 0.90 | [0.47-1.74] |  | 0.87 | [0.45-1.68] |  | 0.88 | [0.46-1.70] |  |
| Alcohol use disorders | 87 | 4 | 4.6 | 0.80 | [0.26-2.44] |  | 0.90 | [0.29-2.78] |  | 0.85 | [0.27-2.65] |  | 0.85 | [0.27-2.65] |  | 0.81 | [0.26-2.54] |  |
| Effort-reward imbalance ratio >1 |  |  |  |  |  | 0.068 |  |  | 0.104 |  |  | 0.142 |  |  | 0.109 |  |  | 0.140 |
| No | 1,256 | 61 | 4.9 | 1 |  |  | 1 |  |  | 1 |  |  | 1 |  |  | 1 |  |  |
| Yes | 1,024 | 68 | 6.6 | 1.39 | [0.98-1.99] |  | 1.35 | [0.94-1.93] |  | 1.31 | [0.91-1.88] |  | 1.34 | [0.94-1.92] |  | 1.31 | [0.91-1.88] |  |
| Biomechanical wrist exposure |  |  |  |  |  | **<0.001** |  |  |  |  |  | **<0.001** |  |  |  |  |  |  |
| No | 895 | 25 | 2.8 | 1 |  |  |  |  |  | 1 |  |  |  |  |  |  |  |  |
| Yes | 1,385 | 104 | 7.5 | 2.82 | [1.81-4.41] |  |  |  |  | 2.72 | [1.74-4.25] |  |  |  |  |  |  |  |
| Chemical exposure |  |  |  |  |  | **0.008** |  |  |  |  |  |  |  |  | **0.013** |  |  |  |
| No | 2,09 | 110 | 5.3 | 1 |  |  |  |  |  |  |  |  | 1 |  |  |  |  |  |
| Yes | 190 | 19 | 10.0 | 2.00 | [1.20-3.34] |  |  |  |  |  |  |  | 1.93 | [1.15-3.23] |  |  |  |  |
| Biomechanical-chemical co-exposure |  |  |  |  |  | **<0.001** |  |  |  |  |  |  |  |  |  |  |  | **<0.001** |
| No exposure group | 861 | 22 | 2.6 | 1 |  |  |  |  |  |  |  |  |  |  |  | 1 |  |  |
| Chemical exposure group | 1,229 | 88 | 7.2 | 3.69 | [1.05-12.99] |  |  |  |  |  |  |  |  |  |  | 3.55 | [1.00-12.62] |  |
| Biomechanical exposure group | 34 | 3 | 8.8 | 2.94 | [1.83-4.73] |  |  |  |  |  |  |  |  |  |  | 2.83 | [1.76-4.57] |  |
| Co-exposure group | 156 | 16 | 10.3 | 4.36 | [2.23-8.50] |  |  |  |  |  |  |  |  |  |  | 4.12 | [2.10-8.07] |  |
| OR: odds-ratio; 95% CI: 95% confidence interval; BMI: body mass index. | | | | | | | | | | | | | | | | | | |
| ^a^ Model 1: Including personal and medical risk factors. | | | | | | | | | | | | | | | | | | |
| ^b^ Model 2: Biomechanical wrist exposure added to model 1. | | | | | | | | | | | | | | | | | | |
| ^c^ Model 3: Chemical exposure added to model 1. | | | | | | | | | | | | | | | | | | |
| ^d^ Model 4: Exposure to Biomechanical wrist stressors and chemical exposure including a co-exposure group added to model 1. | | | | | | | | | | | | | | | | | | |
| ^e^ In bold, p < 0.05. | | | | | | | | | | | | | | | | | | |
